# Inflammatory mediator release from primary rhesus microglia in response to *Borrelia burgdorferi* results from the activation of several receptors and pathways

**DOI:** 10.1186/s12974-015-0274-z

**Published:** 2015-03-28

**Authors:** Geetha Parthasarathy, Mario T Philipp

**Affiliations:** Division of Bacteriology and Parasitology, Tulane National Primate Research Center, Tulane University, 18703, Three Rivers Road, Covington, LA 70433 USA

**Keywords:** *B. burgdorferi*, Non-human primates, Microglia, Inflammation, Toll-like receptors

## Abstract

**Background:**

In previous studies, neurons were documented to undergo apoptosis in the presence of microglia and live *Borrelia burgdorferi*, but not with either agent alone. Microscopy showed that several Toll-like receptors (TLRs) were upregulated in microglia upon *B. burgdorferi* exposure. It was hypothesized that the inflammatory milieu generated by microglia in the presence of *B. burgdorferi* results in neuronal apoptosis and that this inflammation was likely generated through TLR pathways.

**Methods:**

In this study, we explored the role of several TLR and nucleotide-binding oligomerization domain containing 2 (NOD2)-dependent pathways in inducing inflammation in the presence of *B. burgdorferi*, using ribonucleic acid interference (RNAi) and/or inhibitors, in primary non-human primate (NHP) microglia. We also used several inhibitors for key mitogen-activated protein kinase (MAPK) pathways to determine the role of downstream pathways in inflammatory mediator release.

**Results:**

The results show that the TLR2 pathway plays a predominant role in inducing inflammation, as inhibition of TLR2 with either small interfering RNA (siRNA) or inhibitor, in the presence of *B. burgdorferi*, significantly downregulated interleukin 6 (IL-6), chemokine (C-X-C) motif ligand 8 (CXCL8), chemokine (C-C) motif ligand 2 (CCL2), and tumor necrosis factor (TNF) production. This was followed by TLR5, the silencing of which significantly downregulated IL-6 and TNF. The role of TLR4 was inconclusive as a TLR4-specific inhibitor and TLR4 siRNA had opposing effects in the presence of *B. burgdorferi*. Silencing of NOD2 by siRNA in the presence of *B. burgdorferi* significantly upregulated IL-6, CCL2, and TNF. Downstream signaling involved the adaptor molecule myeloid differentiation primary response 88 (MyD88), as expected, as well as the MAPK pathways, with extracellular signal-regulated kinase (ERK) being predominant, followed by Jun N-terminal kinase (JNK) and p38 pathways.

**Conclusions:**

Several receptors and pathways, with both positive and negative effects, mediate inflammation of primary microglia in response to *B. burgdorferi*, resulting in a complex, tightly regulated immune network.

## Background

Lyme disease, caused by the spirochete *Borrelia burgdorferi*, is the leading cause of vector-borne illness in the northern hemisphere, including in the United States [1,2]. Transmitted through the bite of an infected tick, the bacteria disseminates to distal areas, resulting in carditis, uveitis, arthritis, and/or neuroborreliosis of the central and/or peripheral nervous systems (CNS, PNS) [3-6]. Lyme neuroborreliosis, or LNB, is the most morbid form of Lyme disease, due to its at times debilitating and long-lasting neurological sequelae. Signs and symptoms of CNS/PNS disease include meningitis, cranial neuritis, encephalopathies, and encephalomyelitis (rarely), as well as radiculoneuropathy and limb pain or sensory loss [7]. While the type of neurological manifestation varies between patients, a common occurrence is the presence of inflammation, both in LNB as well as in other forms of Lyme disease. The presence of cerebrospinal fluid pleocytosis and perivascular cell infiltrates local to peripheral nerves, brain, and meninges, and other tissues have been recorded in animal models of Lyme disease as well as in human patients [8-10]. Such responses are often accompanied by the production of inflammatory mediators such as interleukin 1-beta (IL-1β), interleukin 6 (IL-6), interferon-gamma (IFNγ), tumor necrosis factor (TNF), chemokine (C-C) motif ligand 2 (CCL2), chemokine (C-X-C) motif ligand 8 (CXCL8), and several others, secreted both by resident cells as well as incoming cells of the immune system [11-15]. Manifestation of such reactive inflammation has often been shown to play a key role in several CNS pathologies such as CNS injury and neurodegenerative diseases [16-19]. By analogy, we hypothesized that such an inflammatory process in the CNS as caused by *B. burgdorferi* plays a role in neuronal and/or glial cell dysfunction, leading to cell loss through apoptosis, and would underlie the pathogenesis of LNB. In evidence, several studies from our laboratory have shown that exposure of brain cells, sections, or tissues to *B. burgdorferi*, be it *ex vivo*, *in vivo*, or *in vitro*, results in production of inflammatory mediators such as IL-6, CCL2, CXCL8, and others and is often accompanied by apoptosis of neurons or oligodendrocytes [20-22]. *In vitro* studies have shown that neuronal loss can occur in the presence of *B. burgdorferi* and microglia, a leading contributor of inflammatory mediators in the CNS, while oligodendrocytes were susceptible to *B. burgdorferi* alone and required no other cell involvement to undergo apoptosis [20,23]. However, as with neurons, oligodendrocyte cell loss occurred within an inflammatory milieu. Furthermore, application of an anti-inflammatory drug mitigated this effect *in vitro* in oligodendrocytes [20], confirming our hypothesis that inflammation contributes to CNS pathology due to *B. burgdorferi*.

Currently, several studies are being undertaken in our laboratory to delineate the molecular mechanisms leading to inflammatory mediator release from the resident cells of the CNS when exposed to *B. burgdorferi*. The hypothesis is that determination of inflammatory mechanisms will also lead to elucidation of cell death mechanisms and thereby provide additional targets of intervention. In a recent study, we have shown that the mitogen-activated protein kinase (MAPK) pathways, particularly the MAP kinase kinase (MEK)/extracellular signal-regulated kinase (ERK) pathway, play a prominent role in the production of inflammatory mediators from oligodendrocytes as well as apoptosis of these cells [24]. In a previous study, a role for Toll-like receptors was indicated when suppression of the TLR2 mRNA through small interfering RNA (siRNA) technology reduced inflammatory mediator production in THP1 monocytes in response to *B. burgdorferi* [25]. Upregulation of TLR2, TLR4, and TLR5 receptors was also seen in primary microglia upon exposure to the bacteria [26]. In this current study, we expand this observation by delineating a role for such receptors as well as nucleotide-binding oligomerization domain containing 2 (NOD2), and for associated MAPK pathways in chemokine and cytokine production by primary non-human primate microglia, in response to *B. burgdorferi*. We show using an additional set of animals to those used in prior publications that the net production of inflammatory mediators is through positive and negative regulation by multiple microglial receptors and pathways.

## Methods

### Bacterial strain and culture

*B. burgdorferi* strain B31 (clone 5A19) was used throughout the course of this study. The strain was routinely cultured in Barbour-Stoenner-Kelly (BSK-H) medium (Sigma-Aldrich, St. Louis, MO) with 0.25 μg/mL amphotericin, 193 μg/mL phosphomycin, and 45.4 μg/mL rifampicin for about 5 to 6 days under microaerophilic conditions. Bacterial concentration was determined using a dark field microscope, and the required number of bacteria was harvested by centrifugation at 2,095 × *g* for 30 min at room temperature, without brakes. The resulting bacterial pellet was resuspended in DMEM:F12 (Invitrogen/Life Technologies, Inc., Grand Island, NY) supplemented with 10% fetal bovine serum (FBS) to the same concentration prior to pelleting and diluted further to the required multiplicity of infection (MOI).

### Isolation and culture of primary microglia

Microglial cells were isolated from frontal cortex tissues of rhesus macaque (*Macaca mulatta*) brains according to previously published protocols [23]. The tissues were obtained from uninfected animals from the breeding colony that were euthanized due to persistent diarrhea or injury. Euthanasia was performed according to the protocols recommended and approved by the Tulane Institutional Animal Care and Use Committee. The microglial isolation protocol was as follows. The leptomeninges and leptomeningeal blood vessels were removed first, followed by mechanical mincing of the cortex tissue with scalpels. The minced tissue was enzymatically digested with 0.25% Trypsin-EDTA containing 200 Kunitz unit/mL DNaseI (Sigma-Aldrich, St. Louis, MO) at 37°C for 20 min with occasional shaking. This enzymatically dissociated tissue was then centrifuged (335 × *g*, 10 min), and the upper layer of cells was removed and filtered through a 20- to 30-μm Nitex filter. The filtrate was resuspended in DMEM:F12 supplemented with 10% FBS, 1% penicillin-streptomycin, and 0.5 ng/mL granulocyte-macrophage colony-stimulating factor (GM-CSF) and seeded as aggregate cultures in T-75 flasks, with the medium changed every 4 days for about 4 weeks. Microglial cells were dislodged from the aggregate cultures by vigorous tapping on the sides of the culture flasks, collected and seeded at the required density. All assays were conducted within 2 to 3 days after seeding of cells. The purity of the microglial culture was determined by staining with antibody to the Iba1 phenotypic marker (Wako Chemicals USA, Richmond, VA) and was assessed to be between 92% and 95%. A total of nine animals were used in this study.

### Infection assays with receptor/pathway inhibitors

Assays were carried out with approximately 1 to 2 × 10^4^ cells/well of microglia seeded in 24-well tissue culture plates. At the time of the assay, the culture medium was removed and replaced with fresh medium without antibiotics, and the cells were pretreated with the pertinent inhibitors for 2 h prior to adding *B. burgdorferi* (MOI of 10:1). The microglial cells were incubated with the bacteria and inhibitors for a further 24 h, followed by collection of supernatant after centrifuging at 2,095 × *g* for 10 min at 4°C. Supernatants were stored at −20°C until analysis. The following inhibitors were used: SB203580 and BIRB796 (p38); U0126 (MEK1/2); SP600125 and Jun N-terminal kinase (JNK) inhibitor VIII (JNK) (all but one were from EMD Millipore, Billerica, MA; BIRB796 was obtained from Cayman Chemical Co., Ann Arbor, MI); and oxidized 1-palmitoyl-2-arachidonoyl-sn-glycero-3-phosphorylcholine (OxPAPC) (TLR2/4), CLI-095 (TLR4), Gefitinib (RipK2), and myeloid differentiation primary response 88 (MyD88) inhibitory peptide (InvivoGen, San Diego, CA).

Pam3CSK4 (Imgenex, San Diego, CA), LPS O55:B5 (Sigma-Aldrich, St. Louis, MO), or muramyl dipeptide (MDP, InvivoGen) were included as positive control agonists when required.

### RNAi

Gene silencing of specific receptors or adaptor molecules was carried out using siRNA technology, according to Dennis *et al*. [25]. Briefly, approximately 2 × 10^4^ microglia/well were seeded in 24-well plates for 48 h, and culture medium replaced with 100 μL antibiotic-free medium. Transfection complexes were generated using 2 μL HiPerfect transfection reagent (Qiagen, Valencia, CA) and 15 to 30 nM siRNA (Santa Cruz Biotechnology, Dallas, TX) in antibiotic- and serum-free medium. The complexes were incubated at room temperature for 30 min and 100 μL of the complex was added to cells. After a 6-h incubation at 37°C and 5% CO_2_, 400 μL of antibiotic-free medium was added and further incubated for 18 h, followed by addition of *B. burgdorferi* (MOI 10:1) or receptor-specific positive controls. After a 24-h incubation with bacteria or controls, supernatants were collected as before and analyzed for chemokine and cytokine expression. A non-specific control siRNA was used as negative control for all experiments. In addition to the positive controls mentioned previously, FliC (from *Salmonella typhimurium*, Enzo Lifesciences, Farmingdale, NY) was used as a TLR5-specific control when required.

To determine the efficacy of TLR2 and NOD2 siRNA, 4-well chamber slides were seeded as before and experimentation was carried out as described previously, with *B. burgdorferi* and MDP as treatment groups, respectively. Processing of chamber slides for immunofluorescence was carried out according to previously published protocols [24]. Rabbit polyclonal primary antibody (1:40 titer) was used for identifying TLR2 (Santa Cruz Biotechnology, Dallas, TX), while a mouse monoclonal was used for NOD2 (1:50; Cayman Chemical Co., Ann Arbor, MI). Appropriate secondary antibodies conjugated to Alexa 488 (1:800 titer) were used to visualize the receptors through fluorescent microscopy.

### Microscopy

Receptor-positive cells were visualized using a Leica DMRE fluorescent microscope (Leica microsystems, Buffalo Grove, IL) and Lumencor SOLA GUI software (Lumencor, Beaverton, OR). Percentage of cells positive for receptors in each treatment group was calculated and graphed using Microsoft Excel®. Approximately 15 to 20 fields were counted for each chamber, and when required, cells were imaged using Nuance Multispectral Imaging System (CRi, PerkinElmer, Waltham, MA). Adobe® Photoshop CS6 software was used to assemble the images.

### Cytotoxicity assay

The cytotoxicity of various concentrations of siRNA and inhibitors on microglia was assayed using tetrazolium dye 3-(4,5-dimethylthiazol-2-yl)-2,5-diphenyltetrazolium bromide (MTT) as a substrate and conducted according to the manufacturer’s protocols (Sigma-Aldrich, St. Louis, MO). Briefly, 5 mg/mL MTT was added to cells and incubated at 37°C and 5% CO_2_ for 2 h. Volume of MTT added corresponded to 10% of the volume of the medium in the wells. After 2 h, the cells were solubilized using the solubilization solution provided and the resulting colored solution was read at 570 nm. The results were normalized to cells treated with either control siRNA or cells with no inhibitors.

### Quantitation of chemokines and cytokines

Enzyme-linked immuno-sorbent assays (ELISAs) for IL-6, CXCL8, CCL2, and TNF were carried out using custom non-human primate-Multiplex kits from EMD Millipore (Billerica, MA) at the Pathogen Detection and Quantification Core, Tulane National Primate Research Center, and performed according to the manufacturer’s protocols. An additional 27-plex human chemokine/cytokine kit (BioRad, Hercules, CA) was used for exploratory experiments or to obtain preliminary data. The results were graphed using Microsoft Excel® and figures were assembled using Microsoft Powerpoint® and Adobe® Photoshop CS6.

### Statistics

Statistical significance was determined using Student’s *t*-test, with values being in duplicate for each analysis. The results were considered significantly different if the probability values (*P*) were <0.05.

## Results

### Role of microglial Toll-like receptors and adaptor molecules in *B. burgdorferi*-induced inflammation

In previous studies, a role for Toll-like receptors (TLRs), particularly TLR2, 4, and 5, in mediating inflammation in microglia in response to *B. burgdorferi* was indicated by the upregulation of these receptor proteins, as evidenced through confocal microscopy [26]. In the present study, a more direct evidence for such a role was sought with the help of TLR-specific siRNA and/or inhibitors, when these were available. siRNA was used at a concentration of 25 nM unless otherwise stated. The toxicity of transcription complexes at higher concentrations (>50 nM) was tested with an MTT-based assay and varied between animals. Hence, these data are not shown here. Lower concentrations of siRNA (e.g., 25 nM) were also tested for toxicity by the MTT-based assay and were deemed non-toxic.

**Figure 1:**
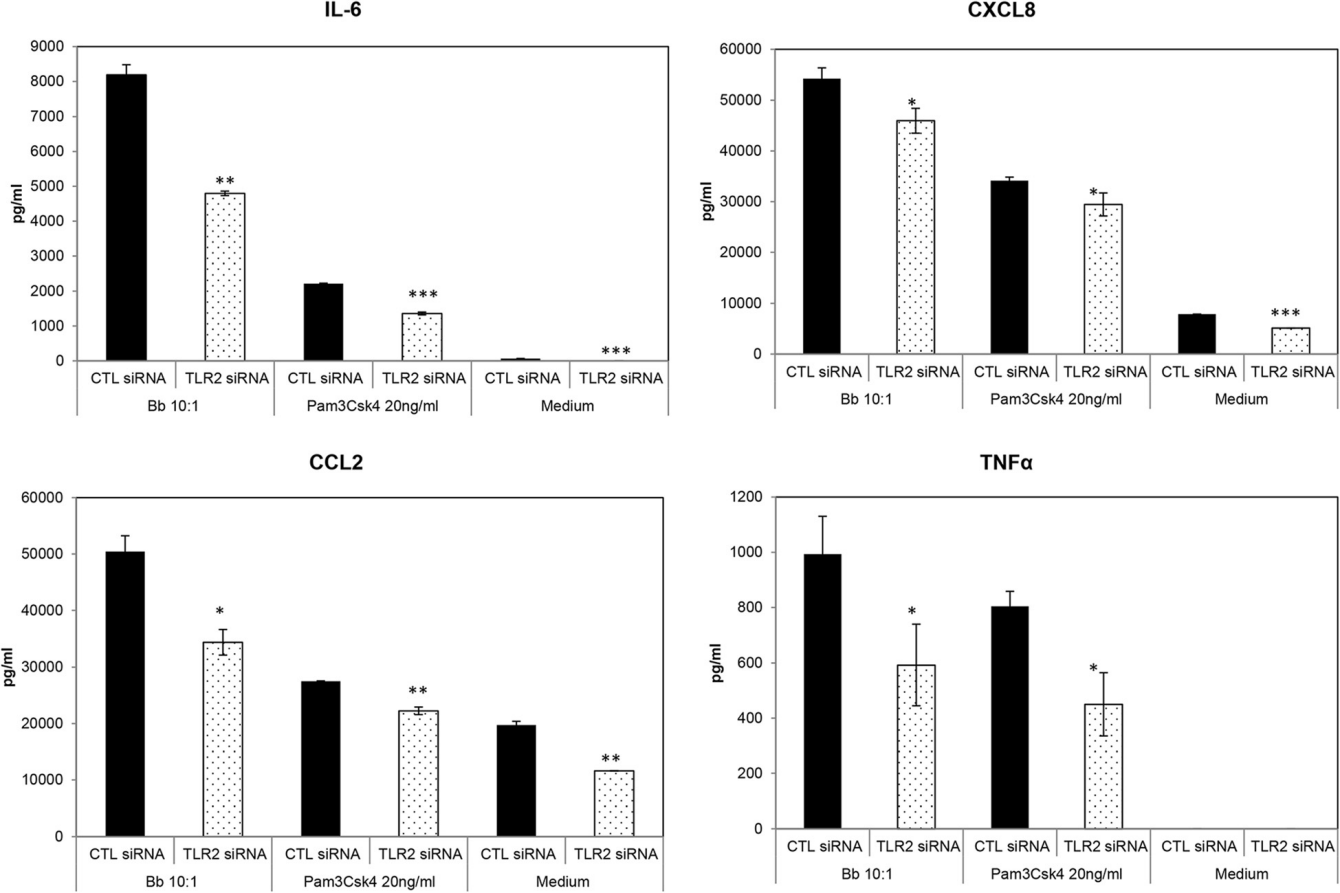
**Role of microglial TLR2 receptor in mediating innate immune responses to** ***B. burgdorferi***. Primary microglia were incubated with 25 nM TLR2 or a non-specific control (CTL) siRNA (described in the ‘Methods’ section) followed by infection with *B. burgdorferi* for 24 h. Supernatants were collected at 24 h post addition of the spirochetes and assayed for cytokine and chemokine levels by a multiplex ELISA. Data are representative of three experiments using microglia from two animals. Pam3CSK4 was used as a positive control and cell culture medium as the negative control. Bars represent standard deviation. *P < 0.05; **P < 0.01; ***P < 0.001. CCL2, chemokine (C-C) motif ligand 2; CXCL8, chemokine (C-X-C) motif ligand 8; IL-6, interleukin 6; siRNA, small interfering RNA; TLR, Toll-like receptor; TNF, tumor necrosis factor.

**Figure 2:**
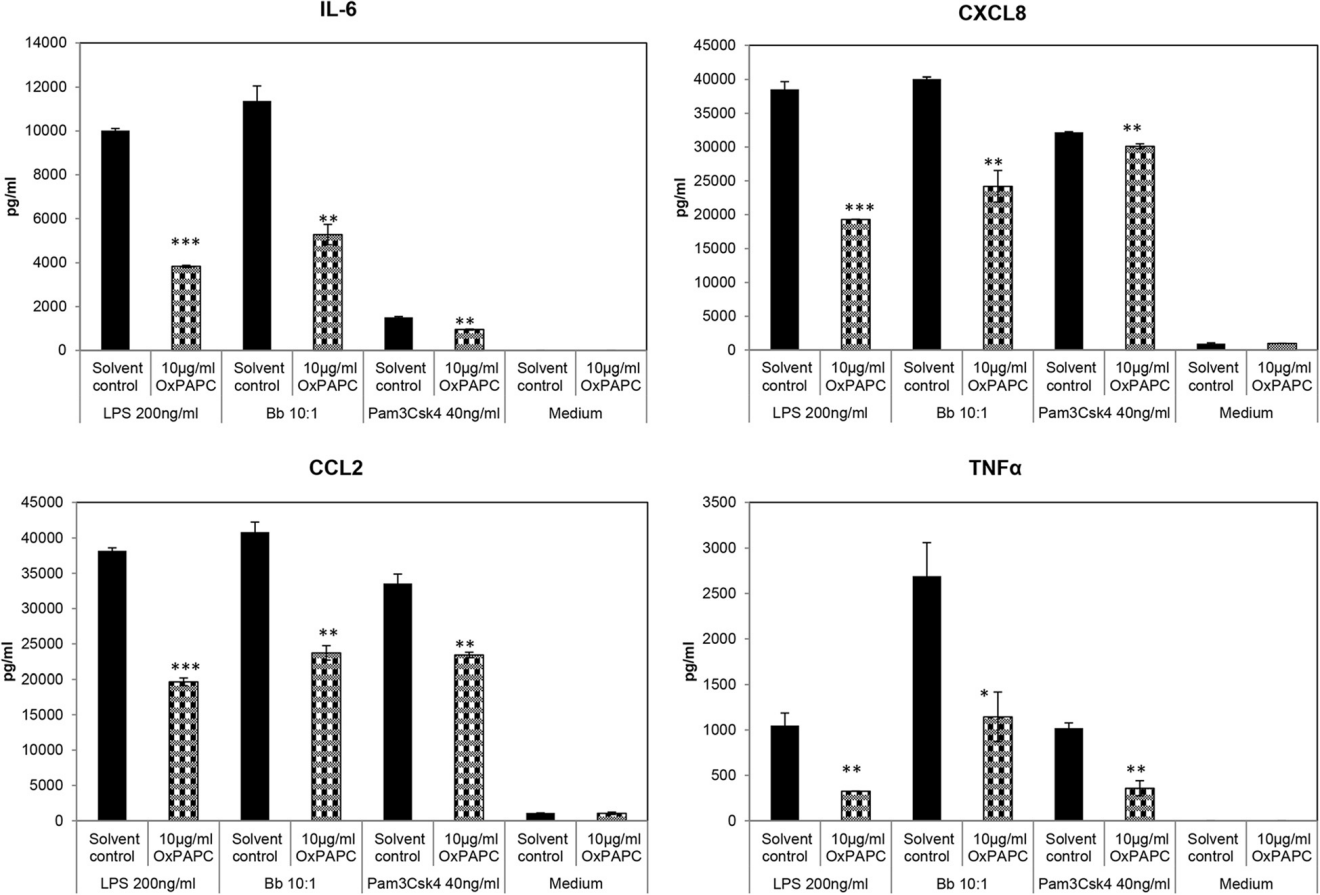
**Effect of a TLR2**/**4 inhibitor on inflammation induced by** ***B. burgdorferi*** **in microglia**. Primary microglia were pretreated with 10 μg/mL OxPAPC, a TLR2/4 inhibitor, for 2 h, followed by *B. burgdorferi* for an additional 24 h. The inhibitor was co-incubated with the bacterium for the duration of infection. After 24 h, the supernatants were collected and stored until analysis. Data are representative of three independent experiments with microglia cultured from two animals. LPS and Pam3CSK4 are positive controls for the inhibitor with the medium serving as control for baseline expression. Bars represent standard deviation. *P < 0.05; **P < 0.01; ***P < 0.001. CCL2, chemokine (C-C) motif ligand 2; CXCL8, chemokine (C-X-C) motif ligand 8; IL-6, interleukin 6; LPS, lipopolysaccharide; OxPAPC, oxidized 1-palmitoyl-2-arachidonoyl-sn-glycero-3-phosphorylcholine.

TLR2 emerged as a prominent receptor in inducing inflammation from primary microglia in response to *B. burgdorferi* exposure (Figures 1 and 2). Suppression of TLR2 mRNA through ribonucleic acid interference (RNAi) as well as inhibition of TLR2/4 signaling by OxPAPC (10 μg/mL) in the presence of *B. burgdorferi* significantly downregulated the release of all four inflammatory mediators tested. OxPAPC did not affect the viability of the bacteria and was non-toxic to microglia at this concentration (data not shown). The efficacy of siRNA or the inhibitor was determined through TLR2- and TLR4-specific ligands Pam3CSK4 and LPS, respectively. As seen in Figures 1 and 2, both positive controls worked as expected, indicating that the specificity of the siRNA/inhibitor was on target. However, considering that the suppression of chemokines/cytokines was more effective when stimulating with LPS than with Pam3CSK4, it is likely that the inhibitor is more effective for TLR4 than TLR2, although it does affect both signaling pathways [27]. In addition, the efficacy of TLR2 siRNA was confirmed using immunofluorescence and microscopy, as well. The overall stimulation of TLR2 in the glial cells was low, with only approximately 25% of cells being positive for the receptor in the presence of *B. burgdorferi*, similar to previously published results where up to a 10% stimulation has been observed [26]. In the presence of *B. burgdorferi*, TLR2 siRNA (25 nM) suppressed the percentage of TLR2 positive cells by about 20%, in comparison to cells that received the non-specific control siRNA (data not shown), indicating that, albeit at a low level, RNAi was achieved.

In order to evaluate more precisely if TLR4 played a role in microglial stimulation with *B. burgdorferi*, a TLR4-specific inhibitor, CLI-095, as well as a TLR4 siRNA was used. The inhibitor blocks signaling from the intracellular domain of TLR4 rather than any adaptor molecule associated with TLR4 and has been shown to be specific for this receptor [28]. In contrast to OxPAPC, which inhibits signaling at the extracellular level of TLR2 and TLR4 (by competitive interaction with TLR2/4 accessory proteins such as CD14, LBP, and MD2), inhibition of intracellular TLR4 signaling significantly increased the production of CCL2 (Table 1). The production of IL-6 and CXCL8 also showed an increasing trend when CLI-095 was used in the presence of *B. burgdorferi* although not statistically significant in a majority of experiments. The inhibitor also had no significant effect on TNF secretion from microglia with *B. burgdorferi*. Higher concentrations of CLI-095 were toxic to cells and hence were not tested. Use of TLR4 siRNA (25 to 30 nM) in the presence of *B. burgdorferi* produced opposing results to the inhibitor data. Here, the siRNA was effective in suppressing expression of CXCL8 significantly, while it showed a similar suppressing effect for IL-6 and CCL2 production, an opposing trend to that seen with CLI-095. The siRNA effect on TNF was an increasing trend overall, although again not significant, and again opposite to the data with CLI-095. In contrast to the results with *B. burgdorferi*, both the inhibitor and TLR4 siRNA significantly suppressed the production of IL-6, CXCL8, and TNF in the presence of LPS with no effect on CCL2. If the siRNA effects hold true, then TLR4 contributes only towards CXCL8 production, but if the inhibitor effects are more concrete, then TLR4 is possibly inhibitory. In either case, in light of these results, it is likely that the predominant effect of OxPAPC on microglia in the presence of *B. burgdorferi* (Figure 2) was delivered via TLR2 rather than through TLR4.

**Table 1:** **Average fold change** ^a^ **in chemokine**/**cytokine expression in the presence of various TLR4 inhibitors**

**Cytokine or chemokine**/**treatment**	**IL**-**6**	**CXCL8**	**CCL2**	**TNF**
Bb/CLI-095	0.901	0.977	0.884^c^	2.239
	(±0.112)	(±0.064)	(±0.114)	(±1.518)
LPS/CLI-095	1.705^b^	1.210^b^	0.957	2.330^b^
	(±0.248)	(±0.075)	(±0.061)	(±1.178)
Bb/TLR4 siRNA	1.218	1.261^b^	1.166	0.893
	(±0.109)	(±0.114)	(±0.104)	(±0.463)
LPS/TLR4 siRNA	1.905^b^	1.341^b^	1.075	3.720^b^
	(±0.305)	(±0.081)	(±0.056)	(±1.309)

^a^Fold change values were calculated as *B. burgdorferi* or LPS with solvent alone/*B. burgdorferi* or LPS with 0.5 μg/mL CLI-095 from five experiments for each treatment with microglia from four animals. Similarly, fold change values were calculated as treatments with control siRNA/treatments with 25 to 30 nM TLR4 siRNA, from four experiments from four animals. Values higher than 1.0 represent fold-down regulation of chemokine/cytokine while values less than 1.0 represent an increase. Standard error of the mean (SEM) is indicated within parentheses. ^b^Values that were significantly downregulated in the majority of experiments (≥60%). ^c^A statistically significant increase in expression in those experiments. Bb, *B. burgdorferi*; CCL2, chemokine (C-C) motif ligand 2; CXCL8, chemokine (C-X-C) motif ligand 8; IL-6, interleukin 6; LPS, lipopolysaccharide; siRNA, small interfering RNA; TLR, Toll-like receptor; TNF, tumor necrosis factor.

**Figure 3:**
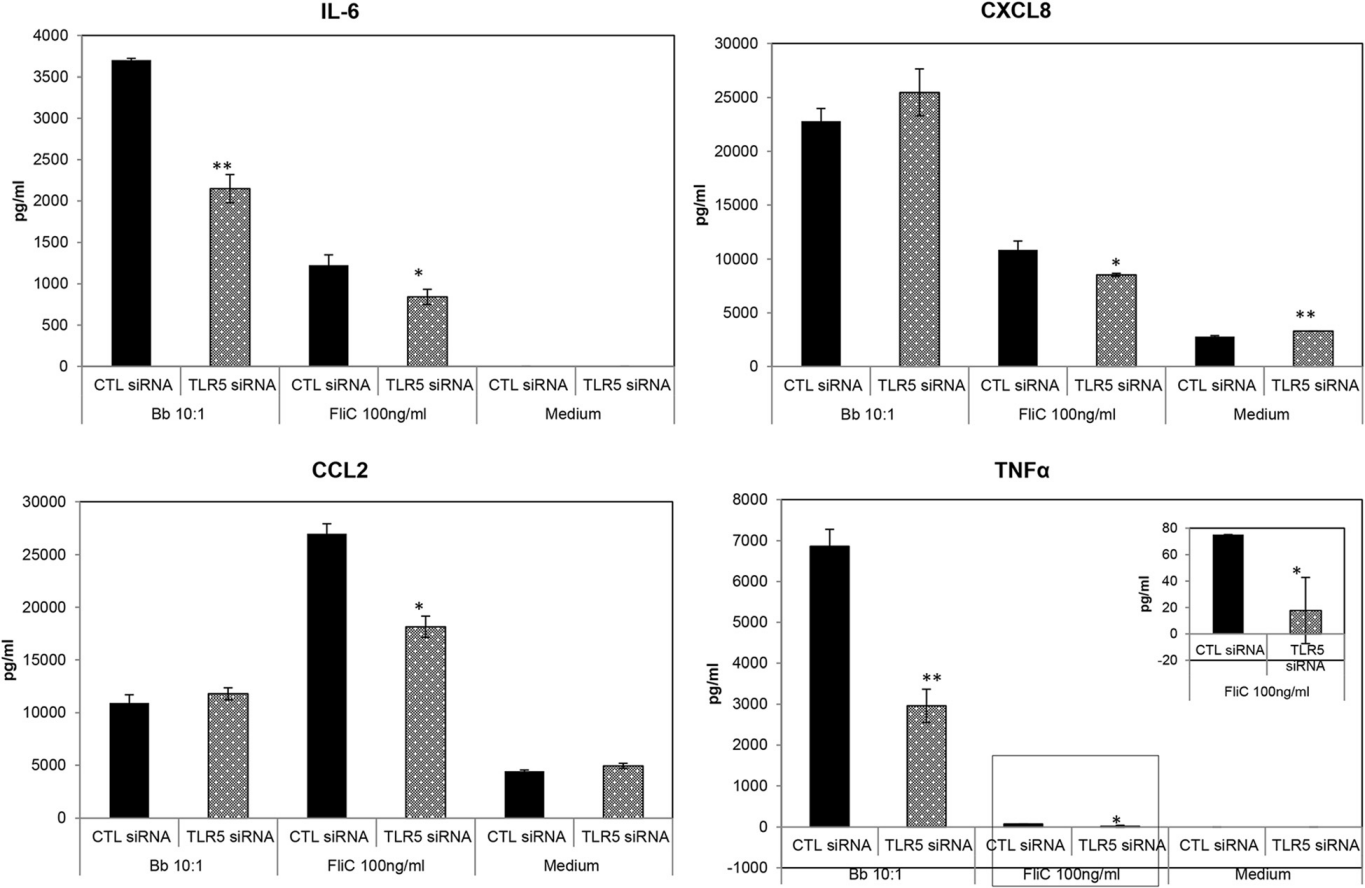
**Role of TLR5 in immune mediator production from NHP microglia in response to** ***B. burgdorferi***. A concentration of 25 nM TLR5 or CTL siRNA was used to determine the role of microglial TLR5 receptor in mediating inflammatory responses to *B. burgdorferi*. A pattern of inhibition was determined from four experiments performed with microglia obtained from two animals. A representative pattern is shown, with bars portraying standard deviation. FliC was used as a positive control for siRNA efficacy, and the medium alone was used as a control for basal expression of inflammatory mediators. *P < 0.05; **P < 0.01; ***P < 0.001. The inset figure shows magnification of the data within the square at the bottom. CCL2, chemokine (C-C) motif ligand 2; CTL, control; CXCL8, chemokine (C-X-C) motif ligand 8; IL-6, interleukin 6; siRNA, small interfering RNA; TLR, Toll-like receptor; TNF, tumor necrosis factor.

As with TLR2, silencing of microglial TLR5 mRNA also suppressed cytokine expression but had no effect on the expression of chemokines by these cells, when siRNA was used at a similar concentration in the presence of the bacteria (Figure 3). In the presence of the positive control FliC; however, TLR5 siRNA did suppress the production of all four mediators tested, indicating that this effect was specific for *B. burgdorferi* and again not due to siRNA defects. There was a small but statistically significant increase in CXCL8 production in the presence of TLR5 siRNA in medium controls. As the tissue culture medium contained serum, which is a complex mix of proteins and other biomolecules, it is conceivable that this small effect was induced through serum components. A similar explanation could also hold true for the low levels of chemokine expression seen with medium-only controls in Figure 1. Since microglia was isolated from animals with injury or persistent diarrhea, medium controls without additional stimulation were included in all experiments to detect baseline inflammatory mediator production. As seen in Figures 1, 2, and 3, regardless of the reason for euthanasia, baseline levels of chemokines and cytokines in microglia were either very low (likely due to serum components) or undetectable.

**Figure 4:**
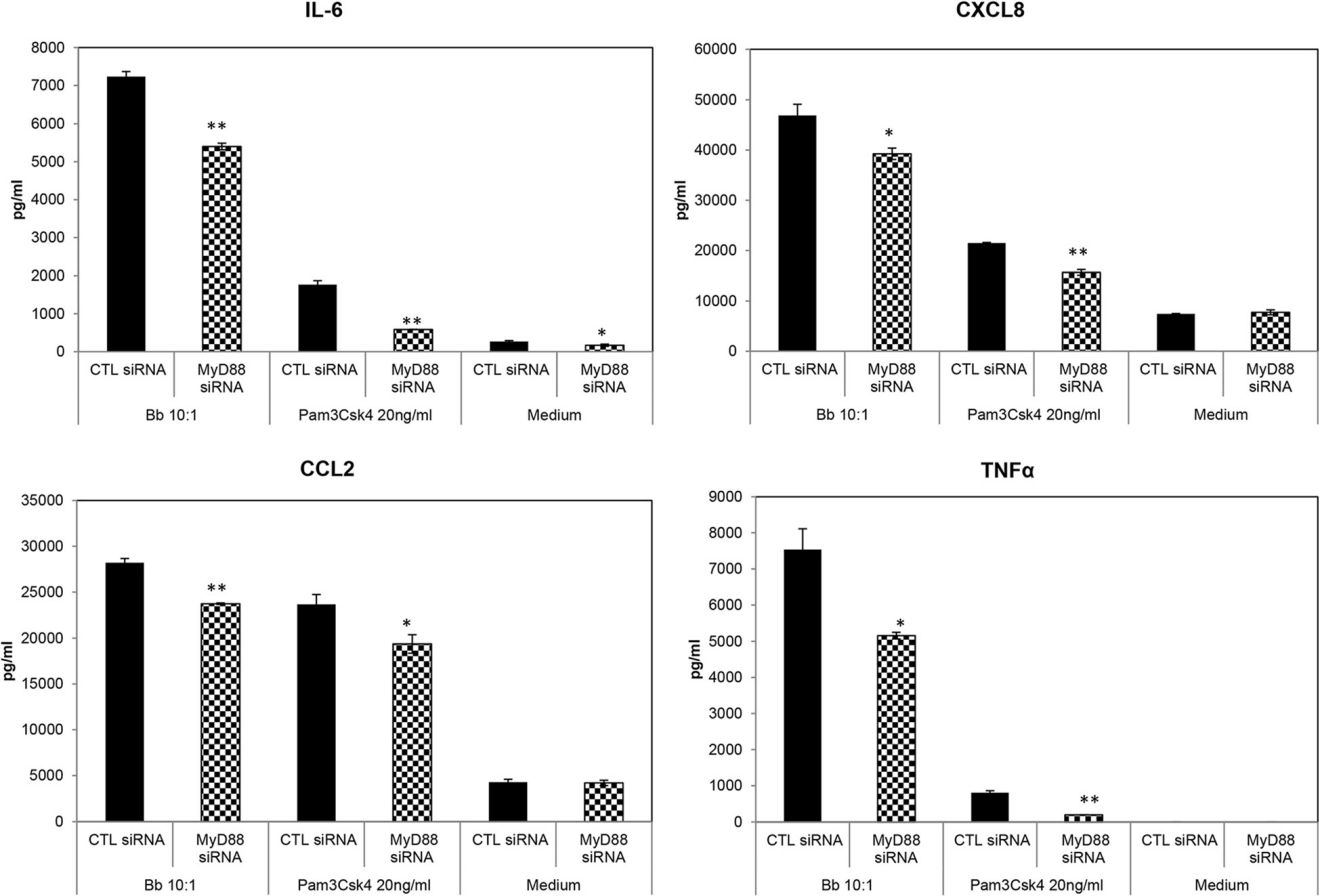
**Intracellular signaling through MyD88.** siRNA for MyD88 (25 nM) was used to determine the role of this adaptor molecule in mediating signaling events in microglia in the presence of *B. burgdorferi*. Data are representative of cytokine/ chemokine pattern as determined from three independent experiments. Bars represent standard deviation. *P < 0.05; **P < 0.01; ***P < 0.001. Pam3CSK4 was used as a positive control for MyD88-mediated effects, and the medium as a control for baseline expression. CCL2, chemokine (C-C) motif ligand 2; CTL, control; CXCL8, chemokine (C-X-C) motif ligand 8; IL-6, interleukin 6; MyD88, myeloid differentiation primary response 88; siRNA, small interfering RNA; TNF, tumor necrosis factor.

As most TLRs signal through the MyD88 adaptor molecule, we next determined the role of this molecule through the use of specific peptide inhibitors and siRNA. However, at low concentrations (≤10 μM), the inhibitor had no conclusive effect while at higher concentrations it was toxic to cells. Hence, further determinations were carried out with siRNA alone. Suppression of MyD88 expression with specific siRNA significantly inhibited the effect of *B. burgdorferi* on the production of all four mediators tested (Figure 4). This also occurred when the stimulant was the TLR2 agonist Pam3CSK4. However, the MyD88 siRNA did not completely abolish mediator production in response to either *B. burgdorferi* or Pam3CSK4, indicating that other adaptor molecules might also play a role, in addition to possible siRNA insufficiency.

### NHP microglial NOD2 receptor plays an anti-inflammatory role in *B. burgdorferi*-mediated inflammatory molecule release

**Figure 5:**
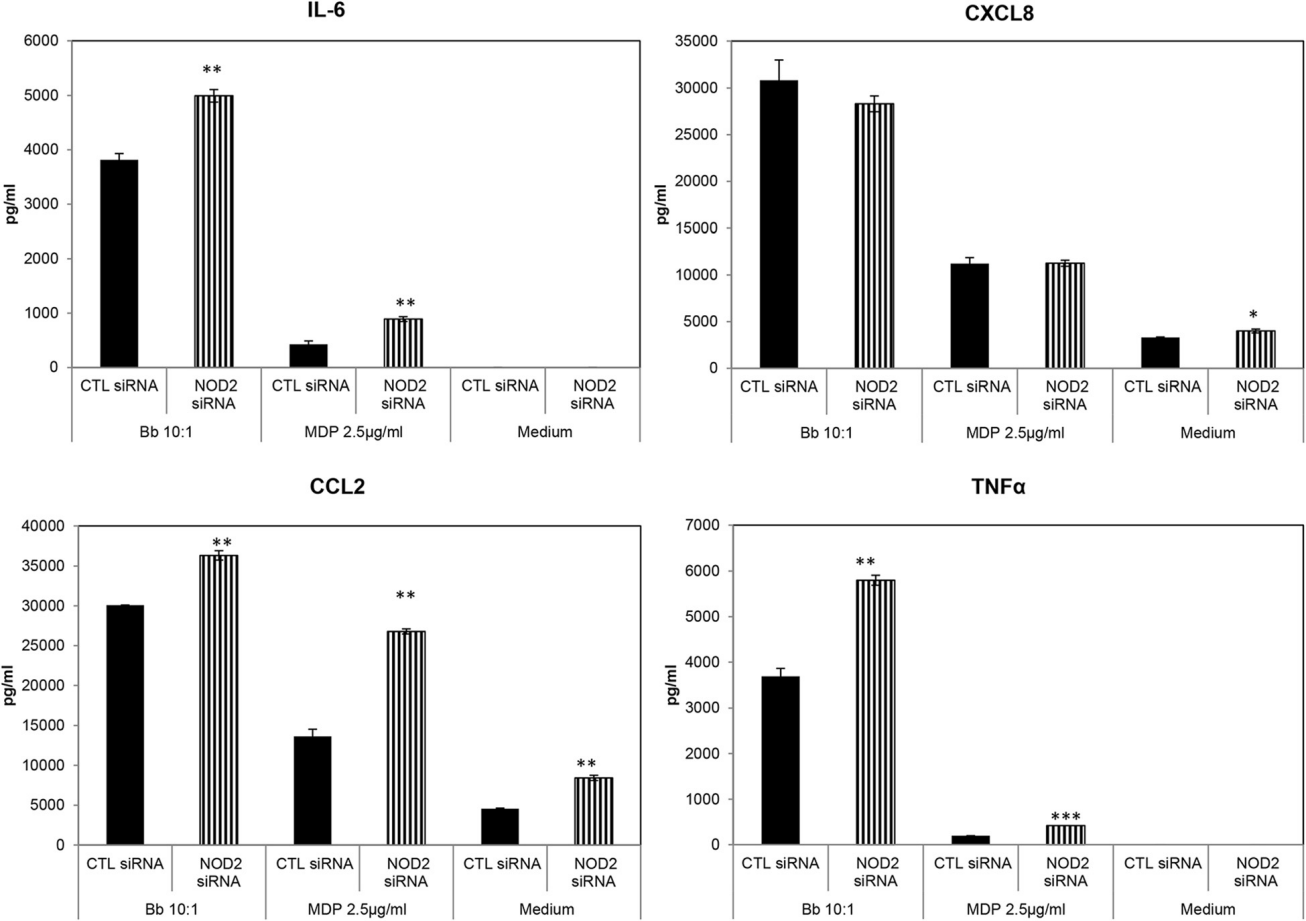
**Role of NOD2 receptor in mediating inflammatory molecule release from microglia exposed to** ***B. burgdorferi***. Primary microglia were co-incubated with 15 nM NOD2 or CTL siRNA for 24 h, followed by *B. burgdorferi* infection for an additional 24 h. Supernatants were collected 24 h post infection and analyzed for chemokine and cytokine expression. A representative pattern from five independent experiments from four animals is shown. Bars represent standard deviation. NOD2 ligand MDP was used as a positive control for NOD2 mediated effects. *P < 0.05; **P < 0.01; ***P < 0.001. CCL2, chemokine (C-C) motif ligand 2; CTL, control; CXCL8, chemokine (C-X-C) motif ligand 8; IL-6, interleukin 6; MDP, muramyl dipeptide; NOD2, nucleotide-binding oligomerization domain containing 2; siRNA, small interfering RNA; TNF, tumor necrosis factor.

**Figure 6:**
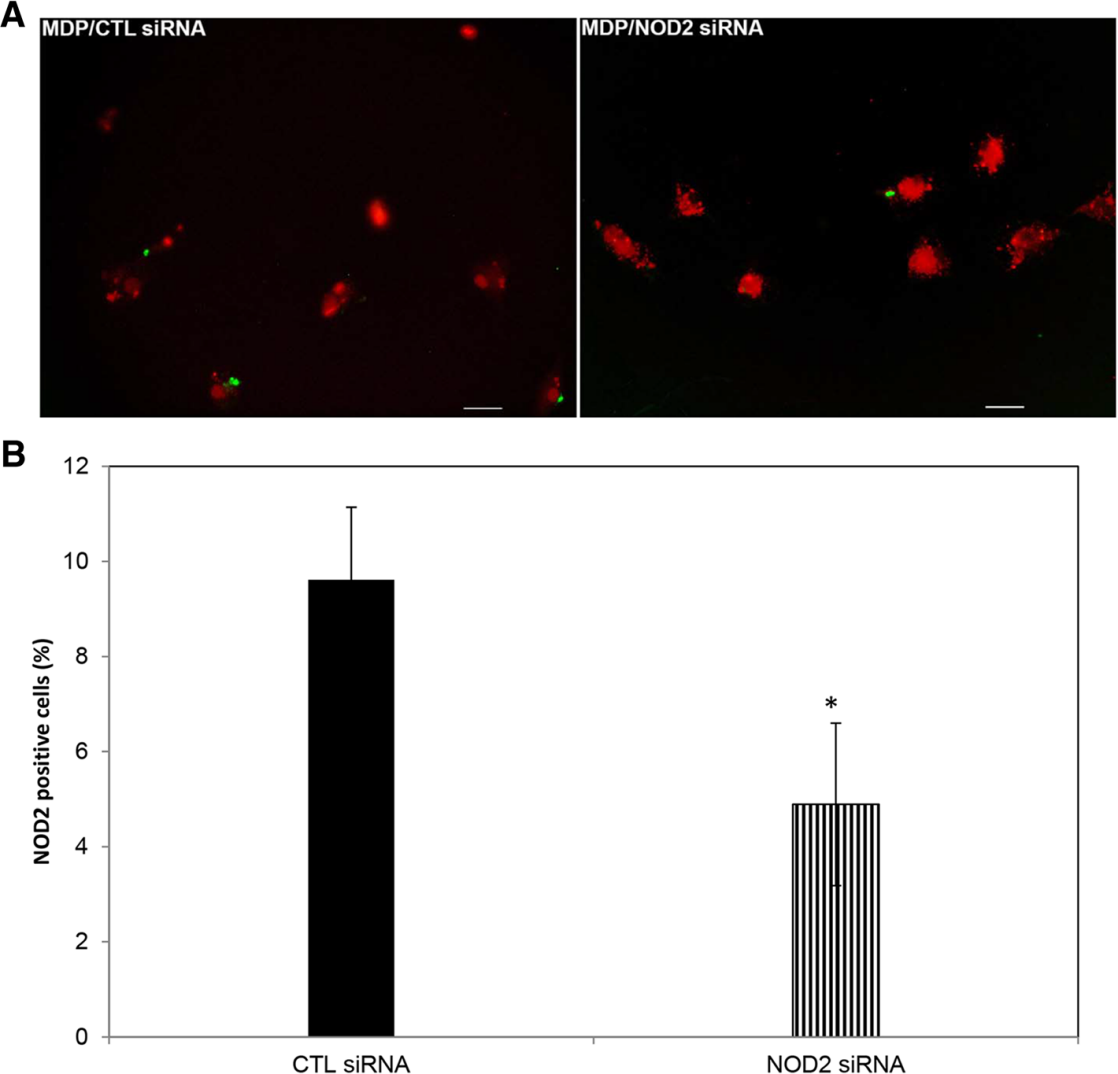
**Efficacy of NOD2 siRNA in suppressing NOD2 protein expression**. RNAi experiment was carried out according to the protocols described in the ‘Methods’ section, with 25 nM control or NOD2 siRNA and 2.5 μg/mL of MDP. Following fixation in paraformaldehyde, cells were analyzed for NOD2 expression by immunofluorescence. **(A)** shows cells (assigned red) stained for NOD2 (green fluorescence) with bars representing 25 μm, and **(B)** shows the percent change in NOD2 expression in the two treatment groups, with vertical bars representing standard deviation. Data was collected from two experiments conducted on microglia collected from a single animal.*P < 0.05. CTL, control; MDP, muramyl dipeptide; NOD2, nucleotide-binding oligomerization domain containing 2; siRNA, small interfering RNA.

NOD2, an intracellular receptor that recognizes muramyl dipeptide (MDP), has been shown to play a role in inflammatory responses to pathogens such as *B. burgdorferi* in mouse astrocytes, mouse microglia, and human peripheral blood mononuclear cells (PBMCs) [29-32]. We sought to determine the role of the non-human primate microglial NOD2 receptor in mediating inflammation that was triggered by exposure to *B. burgdorferi*. MDP was used as a positive control for NOD2 signaling. As seen in Figure 5, suppression of NOD2 mRNA with siRNA (15 nM) in the presence of *B. burgdorferi* resulted in a significantly higher production of chemokines and cytokines with the exception of CXCL8, which remained unaffected. Higher concentrations of siRNA (25 to 30 nM) also showed a similar, significant increase in the production of IL-6, CCL2, and TNF, while it significantly downregulated CXCL8 (Table 2). Silencing of NOD2 expression also generally enhanced the production of pro-inflammatory mediators when the cells were stimulated with MDP (Figure 5 and Table 2), indicating that the NOD2 pathway might be anti-inflammatory in non-human primate (NHP) microglia and might be activated to dampen an otherwise overwhelming immune response that is taking place through other receptors. We tried to confirm these data through use of the receptor-interacting protein 2 (RIP2) tyrosine kinase inhibitor Iressa (Gefitinib, 100 nM). However, Gefitinib, which also inhibits epidermal growth factor receptor (EGFR), had no conclusive effect when used in conjunction with *B. burgdorferi*, but did upregulate CCL2 production on average from four experiments in microglia from two animals (data not shown). Since NOD2 siRNA showed a trend in increasing the production of inflammatory mediators, with both the bacterium and the positive control, we wanted to confirm the efficacy of this treatment. In previous studies [26], it was shown that only about 10% of microglia was activated in the presence of *B. burgdorferi*, and therefore, even though TLR2 receptors were seen to be upregulated microscopically, a quantitative increase in receptor production could not be proven by Western blot. Therefore, we decided to test the efficacy of siRNA microscopically. As seen in Figure 6, we also observed that an approximate 10% of cells stained positive for NOD2 receptor in the presence of positive control MDP (Figure 6B), while it was significantly downregulated in the presence of NOD2 siRNA, to about 5% of cells; a reduction of approximately 50%.

**Table 2:** **Average fold change** ^a^ **in chemokine**/**cytokine expression in the presence of 25** to **30 nM NOD2 siRNA**

**Cytokine or chemokine**/**treatment**	**IL**-**6**	**CXCL8**	**CCL2**	**TNF**
*B. burgdorferi*	0.817^b^	1.165^c^	0.803^b^	0.703^b^
	(±0.041)	(±0.117)	(±0.118)	(±0.116)
MDP	0.7034^b^	1.0295	0.6889^b^	0.6486^b^
	(±0.170)	(±0.115)	(±0.062)	(±0.494)

^a^Fold changes in chemokine/cytokine expression were calculated as treatment with control siRNA/treatment with NOD2 siRNA. Values for *B. burgdorferi* (MOI 10:1) treatment were calculated from three experiments using microglia from three different animals; MDP (2.5 μg/mL) was from four experiments from four animals. Values higher than 1.0 represent fold-down regulation of chemokine/cytokine while values less than 1.0 represent an increase. Standard error of the mean (SEM) is indicated within parentheses. ^b^A statistically significant increase in expression. ^c^Values that were significantly downregulated in the majority of experiments (≥67%). chemokine (C-C) motif ligand 2; CXCL8, chemokine (C-X-C) motif ligand 8; IL-6, interleukin 6; MDP, muramyl dipeptide; TNF, tumor necrosis factor.

### Microglial MAPK pathways are activated during *B. burgdorferi* infection

Signaling through multiple receptors, including TLRs, has been shown to activate MAPK and other pathways, culminating in inflammatory mediator release. In previous studies that focused on oligodendrocytes, when cells were exposed to *B. burgdorferi*, a predominant role for the ERK pathway over other pathways was uncovered [24]. In this study, since TLR pathways were shown to be activated, we evaluated the role of individual MAPK pathways in mediating inflammation due to *B. burgdorferi*. As seen in Table 3, and as with oligodendrocytes, the MEK/ERK pathway, followed by JNK and p38 pathways, played a predominant role in mediating chemokine/cytokine release from primary microglia. At 2.5 μM inhibitor concentration, inhibition of the MEK/ERK pathway suppressed production of all four mediators; this was followed by JNK and p38 suppression, which inhibited the production of four and three mediators each, respectively. The p38 inhibitor had no conclusive effect on CCL2 release. Suppression of NFkB had no conclusive effects in the two animals tested (data not shown). We also tested two other inhibitors, BIRB 796 (p38) and JNK inhibitor VIII, at the same concentration, and found that the overall effect remained unchanged (data not shown).

**Table 3:** **Average fold change** ^a^ **in chemokine**/**cytokine expression in the presence of various MAPK pathway inhibitors**

**Cytokine**/**treatment** (**2.5 μM**)	**IL**-**6**	**CXCL8**	**CCL2**	**TNF**
MEK1/2	*2.639*	*2.215*	*3.813*	*31.546*
	(±0.445)	(±0.226)	(±1.138)	(±18.767)
JNK	*1.475*	*1.760*	*1.512*	*3.991*
	(±0.219)	(±0.202)	(±0.083)	(±0.508)
P38	*1.382*	*1.397*	1.153	*6.301*
	(±0.065)	(±0.057)	(±0.142)	(±2.338)

^a^Fold change values were calculated as *B. burgdorferi* with solvent alone/*B. burgdorferi* with inhibitor from four experiments for each treatment with microglia from two to three animals. Values higher than 1.0 represent fold-down regulation of chemokine/cytokine while values less than 1.0 represent an increase. Standard error of the mean (SEM) is indicated within parentheses. Values that were significantly downregulated in all of the experiments are indicated in italics. CCL2, chemokine (C-C) motif ligand 2; CXCL8, chemokine (C-X-C) motif ligand 8; IL-6, interleukin 6; JNK, Jun N-terminal kinase; MEK, MAP kinase kinase; TNF, tumor necrosis factor.

## Discussion

Toll-like receptors have been shown to play an important role in mediating inflammation in response to a large variety of stimuli. The type and number of TLRs involved depends both on the availability of specific pathogen-associated molecular patterns (PAMP) of the microbe in question, as well as host tissue-specific expression of TLRs, and may coordinate the overall immune response towards that pathogen [33,34]. In previous studies, we have shown via confocal microscopy that the TLR2, 4, and 5 receptors were upregulated in NHP microglia in response to *B. burgdorferi* [26]. TLR2 and 5 were expressed on the cell surface and/or in endosomal compartments in a manner that correlated with the internalization of *B. burgdorferi* by microglia, which is consistent with other studies that showed a role for both TLR transcripts and phagocytosis in the immune response to *B. burgdorferi* [35,36]. Those results indicated that these receptors might be involved in the innate immune response towards *B. burgdorferi* and also that these pathways could play a role in neuronal apoptosis mediated by microglial inflammation [23]. The current study was undertaken to further delineate the role of these and other receptors and pathways in mediating microglial inflammation in response to *B. burgdorferi*, using RNAi technology and other methods. Our results show that TLR2 and TLR5 are both involved in the production of cytokines and chemokines from primary microglia, with TLR2 probably being the predominant TLR (based on the number of mediators that were suppressed by siRNA and inhibitor, from those that were assayed, Figures 1, 2, and 3). This result is in accordance with the presence of TLR2 and TLR5 ligands, namely, lipoproteins and flagellin, respectively, which are present in large amounts in *B. burgdorferi*. The outcome of our TLR experiments is in partial contrast with the results of Dennis *et al*. [25] with THP1 monocytes, wherein TLR2 had a role in mediating inflammation in response to *B. burgdorferi*, but not TLR5. The role of microglial TLR4 in *B. burgdorferi*-mediated inflammation is more ambiguous. *B. burgdorferi* does not possess the classic TLR4 ligand LPS, but may contain a lipooligosaccharide (LOS) moiety [37]. In our previous study [26], this receptor was also upregulated by *B. burgdorferi* concomitantly with TLR2 and 5, perhaps as a result of coordinated regulation without ligand involvement, or through the LOS. In our current study, we found that a specific inhibitor of TLR4 intracellular signaling showed a trend towards increased production of IL-6, CXCL8, and CCL2 in the presence of *B. burgdorferi* (Table 1). This indicated that TLR4 might trigger an inhibitory pathway during exposure to *B. burgdorferi*, coordinately regulated to control excessive cytokine/chemokine production. The effect of TLR4 siRNA, on the other hand, was the opposite. The knock-down of TLR4 suppressed CXCL8 production in microglia and generally showed an opposite effect from that seen with CLI-095 (Table 1). However, both the inhibitor and TLR4 siRNA showed the exact same trend in suppressing IL-6, CXCL8, and TNF production with the classic TLR4 ligand LPS. It is possible that, in the absence of a classic LPS like moiety and therefore a true agonist, the inhibition of TLR4 may have pleiotropic effects. Further experimentation is required for delineation of TLR4 in *B. burgdorferi*-mediated inflammation, which currently remains inconclusive.

Both TLR2 and TLR5 have been shown to signal through the adaptor molecule MyD88 [38,39], and in accordance with these other studies, suppression of MyD88 siRNA did inhibit the production of all four inflammatory mediators tested, in the presence of the bacterium (Figure 4). However, the suppression was not to medium-alone levels, indicating the possibility that the siRNA was insufficient. A higher concentration of siRNA (≥50 nM) was also tested, but due to varying toxicity of the transcription complexes on microglia from different animals, these data have not been presented here. However, as primary cells are hard to transfect, the incomplete blockade of cytokines and chemokines seen in our study is more likely a result of low-transfection efficiencies observed in these cells [40]. Additionally, it is possible that signal transduction is conducted through other molecules such as Toll/interleukin-1 receptor (TIR)-domain-containing adapter-inducing interferon-β (TRIF). Although only few studies exist on the possibility of TLR2- or TLR5-mediated signaling through TRIF [41,42], it is nevertheless a viable alternative in microglia.

NOD2 is an intracellular receptor that signals upon binding of MDP. Studies conducted to date with *B. burgdorferi* have largely shown that this spirochete induces inflammation through this receptor [29-32]. In our study, however, suppression of NOD2 expression with siRNA (verified in Figure 6) showed that, in the presence of *B. burgdorferi*, this resulted in an increase in the production of inflammatory mediators, with the exception of CXCL8 (Figure 5 and Table 2). This partial pattern of upregulation in the presence of NOD2 siRNA was seen in microglia at both the concentrations tested and was also evident with the positive control MDP. At least part of this result with NOD2 siRNA was also obtained with Gefitinib, an inhibitor of RIP2, a kinase that functions downstream of NOD2. Addition of Gefitinib and *B. burgdorferi* also upregulated CCL2 production on average, from four experiments in microglia from two animals (data not shown). In addition, Gefitinib significantly increased the production of MIP-1β in the presence of *B. burgdorferi*, and a similar trend was observed with MIP1α and RANTES production in two experiments (not shown). Although Gefitinib also suppresses EGFR signaling, taking all the data together, it is likely that NOD2 signaling (perhaps together with EGFR) suppresses production of at least a few chemokines and/or cytokines, perhaps to control inflammation mediated through TLRs. In fact, NOD2 has been shown to inhibit TLR2-mediated signaling in other studies [43,44], indicating that this possibility is viable. In one study, when splenocytes from NOD2-deficient mice were stimulated with peptidoglycan (PGN), a TLR2 ligand, the cytokine production was found to be higher than those from wild-type mice [44]. Similarly, when mouse peritoneal macrophages were stimulated with both PGN and MDP, production of IL-1β was significantly lower than when stimulated with PGN alone [43], indicating that NOD2 acts as an inhibitory pathway, downregulating TLR2-mediated responses. Also, our results are somewhat similar to those obtained by Chauhan *et al*. [45] where, in the absence of NOD2, osteoblasts that passively internalized *Staphylococcus aureus* or a non-invasive strain of *Salmonella* produced elevated levels of select cytokines, but not with actively invasive *Salmonella*. Additionally, NOD2-mediated effects might also be dose dependent, as seen in the study by Borm *et al*. [46] where MDP added in low doses to TLR2-primed monocytes augmented cytokine responses while it inhibited them at a higher dose. This indicates that host and type of cell, dose effects, bacterial species, and invasive potential of bacteria and other factors may all play a role in differential NOD2-mediated effects.

MDP is generally known to be a specific ligand for NOD2. However, in our study, in the presence of NOD2 siRNA, levels of most chemokines/cytokines went up significantly, indicating that the basal levels without NOD2 siRNA are likely through other pathways as well [47]. The MDP used in our system is a synthetic form, according to the manufacturer. Therefore, the likelihood of endotoxin or peptidoglycan contamination is very low. But, monoacylated forms of MDP have been shown to trigger TLR2/4 pathways [48]. Whether MDP derivatives are formed in cell culture in the presence of eukaryotic cells like microglia, is not known but is an as yet unproven possibility.

The majority of the studies conducted thus far have implicated downstream MAPK pathways in mediating inflammation in a wide variety of cells in response to TLR triggers [33,34,49]. The analysis we performed in NHP primary microglia with the aid of specific inhibitors showed that, of the three MAPK pathways, the ERK pathway predominates in mediating inflammation, followed by the JNK and p38 pathways (Table 3). This order of relevance is similar to that observed in MAPK pathways of human oligodendrocytes in response to *B. burgdorferi* [24]. Since the specificity of U0126, the MEK1/2 inhibitor, has been shown to be very high for its target [50], we did not use additional inhibitors. However, we did use additional inhibitors for p38 and JNK pathways to reconfirm the pattern of inhibition with these specific MAPK pathways and found them unchanged.

**Figure 7:**
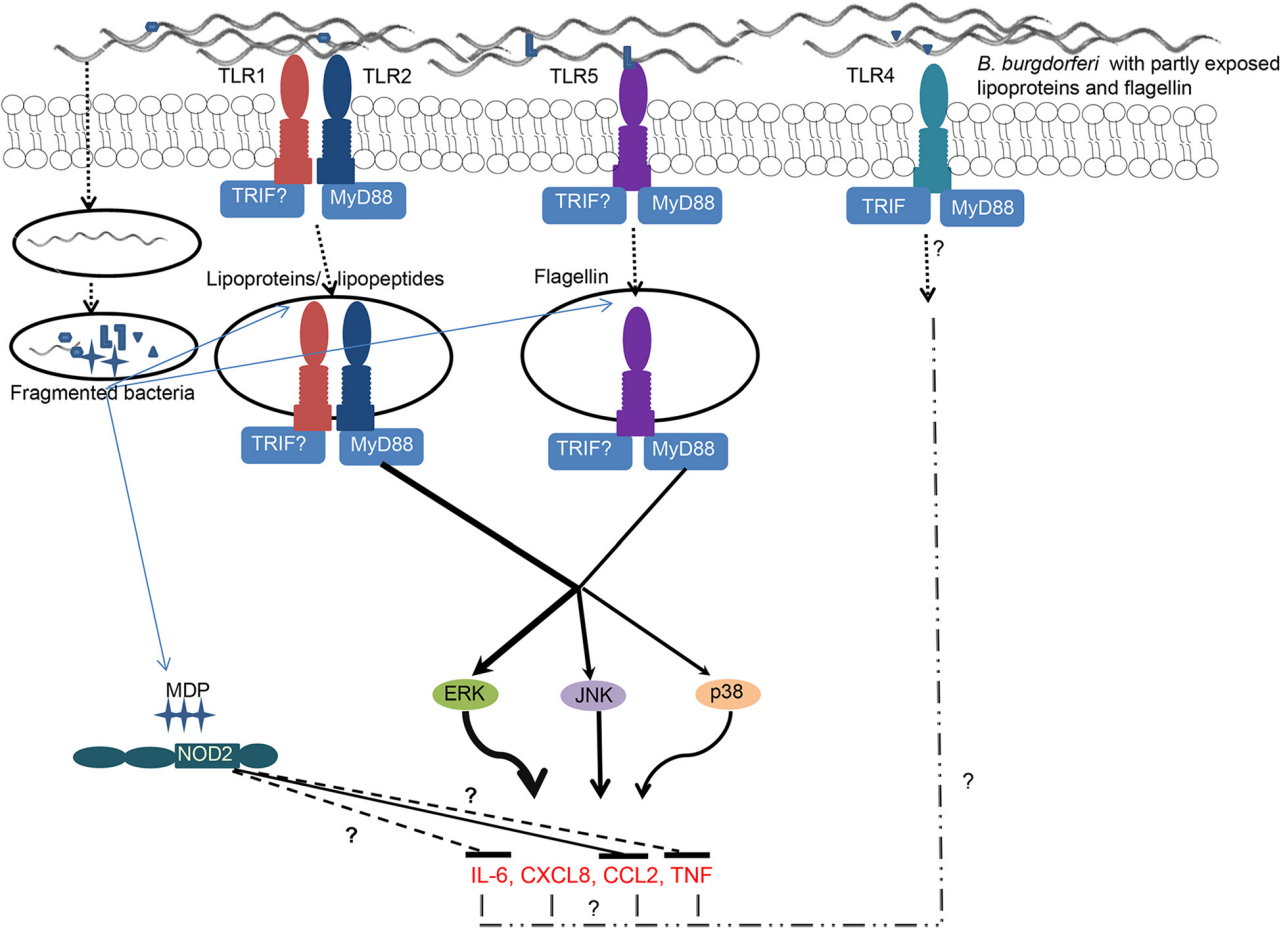
**Proposed model of signaling pathways activated in primary NHP microglia in response to** ***B. burgdorferi***. Upon contact with the bacterium, the microglial TLR2 and TLR5, respectively, sense *B. burgdorferi* lipoproteins and flagella (likely exposed by fragmented or by phagocytosed bacteria), initiating downstream responses by recruitment of adaptor molecules such as MyD88. Whether signaling is initiated both at the surface and in the intracellular compartments or exclusively through the latter is not clear, neither is the mechanism of TLR2/5 internalization. However, once signaling is initiated, further downstream signaling leads to activation of MAPK pathways, mainly the ERK pathway, leading to production of inflammatory mediators. Upregulation of TLR4 has been detected by confocal microscopy, perhaps through a lipooligosaccharide moiety or as a result of concomitant activation along with other TLRs. Its role in inflammation is unclear and neither is its intracellular localization (hence the question mark in the corresponding arrow). Signaling through intracellular NOD2, mediated by MDP molecules in phagocytosed bacteria, leads to anti-inflammatory effects, especially for CCL2 and perhaps other mediators. Thus, the overall inflammatory outcome in microglia in response to *B. burgdorferi* is likely through the positive and negative regulation of several key signaling pathways. Thickness of the downstream signaling lines depicts signal strength. Arrowheads indicate induction, and horizontal bars at the end of the lines indicate inhibition. Dashed lines show additional possible inhibitory signals. Dotted lines indicate internalization either through phagocytosis or endocytosis, with phagosome and/or endosome in open ovals. Dashed and dotted lines indicate unclear processes. CCL2, chemokine (C-C) motif ligand 2; CXCL8, chemokine (C-X-C) motif ligand 8; ERK, extracellular signal-regulated kinase; IL-6, interleukin 6; JNK, Jun N-terminal kinase; MDP, muramyl dipeptide; MyD88, myeloid differentiation primary response 88; NOD2, nucleotide-binding oligomerization domain containing 2; TLR, Toll-like receptor; TNF, tumor necrosis factor; TRIF, Toll/interleukin-1 receptor (TIR)-domain-containing adapter-inducing interferon-β.

We have conducted a comprehensive analysis of the role of receptors and pathways in mediating inflammation in primary NHP microglia in response to *B. burgdorferi*. While we have attempted to provide more than one line of evidence in each case, this was not always possible, either due to lack of availability of certain reagents or their associated toxicity or lack of specificity towards a particular target molecule. However, we do provide sufficient evidence for a picture to emerge on the role of these various receptors and pathways in the response to *B. burgdorferi* and have depicted this in the model shown in Figure 7. We show here that multiple receptors and pathways positively and negatively regulate inflammation in primary microglia. A study of the roles of these inflammatory mechanisms in inducing apoptosis in neurons is currently underway.

## Conclusions

The overall immune response from primary NHP microglia upon exposure to *B. burgdorferi* results from a complex network of multiple receptors and pathways that are triggered by a variety of ligands on the bacterium. The result is a tightly regulated immune network with both positive and negative regulation of immune mediators. The key receptors and pathways that were identified in this study may serve as targets for new therapeutics in CNS Lyme disease.

## Abbreviations

CCL2: Chemokine (C-C) motif ligand 2
CD14: Cluster of differentiation antigen 14
CNS: Central nervous system
CXCL8: Chemokine (C-X-C) motif ligand 8
DMEM: Dulbecco’s Modified Eagle’s Medium
EGFR: Epidermal growth factor receptor
ERK: Extracellular signal-regulated kinase
GM-CSF: Granulocyte-macrophage colony-stimulating factor
IL-1β: Interleukin 1-beta
IL-6: Interleukin 6
IFNγ: Interferon-gamma
JNK: Jun N-terminal kinase
LBP: LPS-binding protein
LNB: Lyme neuroborreliosis
LPS: Lipopolysaccharide
MAPK: Mitogen-activated protein kinase
MD2: Myeloid differentiation 2
MDP: Muramyl dipeptide
MEK: MAP kinase kinase
MTT: 3-(4,5-dimethylthiazol-2-yl)-2,5-diphenyltetrazolium bromide
MyD88: Myeloid differentiation primary response 88
NHP: Non-human primate
NOD2: Nucleotide-binding oligomerization domain containing 2
OxPAPC: Oxidized 1-palmitoyl-2-arachidonoyl-sn-glycero-3-phosphorylcholine
PBMCs: Peripheral blood mononuclear cells
PGN: Peptidoglycan
PNS: Peripheral nervous system
RNAi: Ribonucleic acid interference
siRNA: small interfering ribonucleic acid
TLR: Toll-like receptor
TNF: Tumor necrosis factor
